# LGBTQI+ Healthcare (in)Equalities in Portugal: What Can We Learn from Asexuality?

**DOI:** 10.3390/healthcare9050583

**Published:** 2021-05-14

**Authors:** Rita Alcaire

**Affiliations:** Centre for Social Studies, University of Coimbra, 3000-995 Coimbra, Portugal; ritaalcaire@ces.uc.pt

**Keywords:** asexuality, sexual citizenship, life course inequalities, LGBTQ health, healthcare

## Abstract

The main purpose of this article is to analyse how healthcare providers in Portugal perceive asexuality. To do so, the author makes use of qualitative data from both the CILIA LGBTQI+ Lives project and The Asexual Revolution doctoral research on asexuality in Portugal, namely, a focus group conducted with healthcare providers, drawing from their assessment of interview excerpts with people identifying as asexual. The data were explored according to thematic analysis and revealed three major tendencies: (1) old tropes at the doctor’s office; (2) narratives of willingness to learn about the subject; and (3) constructive and encouraging views of asexuality. From this analysis, valuable lessons can be drawn concerning the respect for gender and sexual diversity. The author argues that both formal and informal learning play an important role in building cultural competence among healthcare providers. This could be achieved both by introducing sexual and gender diversity in curricula in HE and through media exposure on these subjects. Overall, it will lead to building knowledge and empathy about marginalised groups, and will help fight inequalities of LGBTQI+ people in healthcare. As such, LGTBQI+ activism that puts the topics of asexuality and LGBTQI+ in the media agenda, is a powerful strategy. Hence, because healthcare providers show willingness to learn, the media becomes a source for learning about asexual and LGTBQI+ experiences, which they can incorporate in their medical practice.

## 1. Introduction

Access to adequate healthcare is a fundamental right consecrated in international documents, namely the Constitution of World Health Organization which declares that “the enjoyment of the highest attainable standard of health is one of the fundamental rights of every human being without distinction of race, religion, political belief, economic or social condition” [1]. The Constitution of the Portuguese Republic echoes this statement (article 64), by declaring that all have the right to the protection of health and the duty to defend and promote it [2]. Therefore, being lesbian, gay, bisexual, trans, queer, intersex, asexual, or other non-normative identity or orientation (LGBTQI+) cannot represent a barrier in access to healthcare [3]. Nevertheless, academic research and empirical evidence have shown that this is not always the case.

Both CILIA LGBTQI+ Lives [4] (Comparing Intersectional Life Course Inequalities amongst LGBTQI+ Citizens in Four European Countries) and The Asexual Revolution [5] projects have revealed the existence of prejudice and discrimination against LGBTQI+ people in sectors that involve the protection of fundamental human rights. This evidence is in agreement with data from the Council of Europe’s report on discrimination on grounds of sexual orientation and gender identity in Europe [6], which investigated the presence of prejudice by professionals whose role is to promote the wellbeing and the rights of all people and their families, irrespectively of their sexual orientation. Moreover, empirical data have clearly shown that prejudice and discrimination against non-heterosexual people not only persist but have assumed more subtle nuances as opposed to more traditional forms [6,7,8]. In other words, prejudice and discrimination have assumed less evident formats in order to adapt to social contexts that are governed by social norms of equality and respect for diversity [9].

LGBTQI+ people have particular health issues that require attention, but many constraints come from clinical practices that are not specific to their needs and from the lack of, or non-application, of existing laws. At the same time, healthcare professionals have deficient instruction in LGBTQI+ issues, namely, when it comes to advanced training in cultural competence [10,11], an area in dire need of investment.

Data from the Council of Europe [6] identified four main obstacles expressed by LGBTQI+ people in accessing healthcare: (1) high level of suspicion towards healthcare professionals; (2) prejudiced attitudes by healthcare professionals; (3) lack of visitation rights and lack of participation in clinical decisions; and (4) the link of gay and bisexual men to HIV/AIDS. In Portugal, the situation is identical. Doctors and nurses have been perceived as homophobic [12]; an analysis of mental health professionals revealed the belief of homosexuality as a non-natural orientation resulting from a deficit or a developmental flaw [13,14]; with the approval of same sex marriage, married partners are protected, but when one is not legally recognised as father/mother, they still face this issue [9]; and the Portuguese Blood Institute excluded gay men from donating by stating they would be more sexually promiscuous than heterosexual men [15,16]. Regarding healthcare, Portuguese LGBTQI+ people have also reported clear evidence of heterosexism [17] and heteronormativity [18].

Healthcare professionals face several challenges throughout their academic and professional life, such as the duration of their training, the long working hours, and stress of making life or death decisions. One of the most significant demands placed on healthcare professionals is the need to adapt the care they provide. For instance, they must consider their patients’ individual characteristics as well as their historical, social, cultural, and political background [19]. In healthcare higher degree curricula (such as nursing, medicine, psychology, social services), as well as in subsequent advanced professional training, pedagogical opportunities on LGBTQI+ issues are scarce, recent, and some are restricted to those with specific instruction and financial resources. They are usually provided by academics with research on these subjects or members of community-based organisations. The result is an absence of specialised education on sexual diversity that, together with a lack of interpersonal contact with LGBTQI+ people, is directly related to negative attitudes towards this population [19,20]. Learning about specific LGBTQI+ lived experience makes it possible to recognise important intersections of cultural, social, and economic dimensions (such as class, race, education, income) as determinants of health and, at the same time, become aware of beliefs and attitudes towards gender and sexuality that can generate inadequate and discriminatory responses. The result is culturally competent knowledge about LGBTQI+ issues, and consequently the ability to adapt the provided care.

The main purpose of this article was to analyse how healthcare providers in Portugal perceive asexuality. To achieve this aim, information is presented concerning LGBTQI+ people’s perceptions of (in)equalities in the Portuguese healthcare sector from data collected in the CILIA LGBTQI+ Lives project, and then an example of positive outcomes of collective action and media presence of asexually identified people towards contributing to the cultural competence of healthcare providers from the narratives collected in a focus group with healthcare providers is introduced.

The choice of using data from a project on LGBTQI+ life course inequalities and another on asexuality concerns the fact that all the identities in the LGBTQI+ spectrum (asexual or allosexual, a term used most commonly in the asexual community to refer to someone who is not asexual) have followed a similar course in terms of both pathologisation and subsequent contestation of that biomedical framing [21]. Namely, homosexuality was considered a mental illness, trans identities were framed under gender dysphoria, and asexuality as hypoactive desire disorder [22] with subsequent social and political implications, such as allowing for human rights violations and discriminations and lack of legal protection. All three mental health diagnoses were contested both by individuals and by members of the medical community, reclaiming their bodies and their lives [21]. The reframing (in the case of asexuality and trans identities) or the abolition (in the case of homosexuality) of these sexual classifications in the Diagnostic and Statistical Manual of Mental Disorders was a result of direct action and presence in the public sphere, aiming at constructing their own social narratives beyond psychiatric discourses. In that sense, asexuality is very much part of the broader conversation about how gender and sexual diversity is being challenged and disputed in Western society. Additionally, the presence of asexuality in discussions on sexual diversity is important, not only for LGBTQI+ and asexually identified people themselves, but also people that are questioning (as in still exploring one’s sexuality).

The article is divided into five main sections. The next segment is dedicated to the methodology and research design, describing data collection methods for both projects from which this article draws information. I present the key results in Section 3, addressing both the perception on (in)equalities by LGBTQI+ interviewees and the main findings of the focus group with healthcare providers. In the following Section, the author discusses the main results and provides lessons to be drawn from them which contribute to a more inclusive medical practice, and finally, concludes the article by providing some recommendations.

## 2. Materials and Methods

### 2.1. Study Design

This article included qualitative data collected in the scope of CILIA LGBTQI+ Lives, a project in which the author is a Research Fellow for the Portuguese team, and from the author’s doctoral research entitled ‘The Asexual Revolution: discussing human rights through the lens of asexuality in Portugal’.

CILIA LGBTQI+ Lives (2018–2021), is being conducted simultaneously in four European countries: England, Germany, Portugal, and Scotland. In Portugal, the project is being developed at the Centre for Social Studies—University of Coimbra. CILIA LGBTQI+ Lives investigates potential inequalities experienced by lesbian, gay, bisexual, transgender, queer and other gender and sexual dissident people at three transition points in life: school to work transition, employment progression in mid-life, and the transition into retirement. The key objective is to provide cross-cultural evidence concerning life course inequalities experienced by LGBTQI+ people, comparing and contrasting these across the four European countries. CILIA LGBTQI+ Lives uses a mixed methods research design that includes secondary analysis, semi-structured interviews, and agent-based modelling (through a social simulation model), across five work packages.

The Asexual Revolution was aimed at understanding how discourses and practices about asexuality—produced by Portuguese media, healthcare providers and asexually identified people—have been constructed in contemporary Portugal. The data were collected over 24 months and include fieldnotes and participant observation, reports of asexuality related events, media coverage from 2001 to 2017, semi structured interviews with self-identified asexuals (April–November 2016), and a focus group with healthcare providers (February 2017).

### 2.2. Participants

The callout for CILIA LGBTQI+ Lives was made through mailing lists, websites, social networks, NGOs, and a Facebook event. The result was a diverse sample, considering age, gender, sexuality, ethnicity, race, disability, health, religion and spirituality, education, social class, and place of residence.

The focus group conducted for The Asexual Revolution was composed of five participants (three cisgender women and two cisgender men) working either in the Portuguese National Health System and/or private practice. Their ages ranged from 48 to 75 years old. The professional areas represented in this session extended from psychiatry/sexology to psychology/psychoanalysis, primary care, gynaecology/obstetrics, and mental health nursing. All participants held higher education degrees and were considered experts in their areas of knowledge and/or worked in excellence institutions in their areas of expertise. The combination of diverse sociodemographic characteristics of participants aimed to ensure necessary differences throughout the discussion process [23]. The use of sociodemographic variants combined with specific particularities has been considered in the literature on focus groups as pertinent to the study and as a means to enrich results. The purpose was not to investigate the recurring discourses but to obtain the greatest discursive variability; therefore, the criterion of representativeness of several healthcare areas of expertise that engage directly with sexuality issues became the most relevant.

### 2.3. Data Collection

The current article draws on data collection performed in the scope of Work Package 3 of CILIA LGBTQI+ Lives that was designed to acquire information on LGBTQI+ life course (in)equalities at the experiential and a subjective level through in-depth interviews. The interview covered experiences at school and their impact on choosing a professional path; adulthood and workplace experience (discrimination, law and regulations, influence of workplace in personal life, positive experiences such as advising or training co-workers on LGBTQI+ issues); retirement and old age (challenges and opportunities); care networks; identities and intersectionalities; and overall discrimination experiences and perceptions of (in)equalities. The semi-structured interviews were intended to encourage the participants to reflect on their past and their future in a retrospective/prospective way. In other words, younger participants (18–30 years old) were asked about their current experience, but also on plans for their retirement and old age; midlife participants (30–50) were inquired about past and present experiences and ideas about the future; and older participants (55+ years old) were asked to reflect on their previous experiences and to consider their later life. All participants were asked to discuss their perceptions of (in)equalities in Portugal, namely, by focusing on whether or not they felt things were improving for LGBTQI+ people, significant changes they witnessed or experienced throughout their life course, and how it they believe it compares to the context in other countries. Participants were also invited to reflect on social, historical, or political changes for LGBTQI+ people that had the most impact on their lives and envisage what is missing in Portugal for full LGBTQI+ equality. The interview process took place throughout 2019 in all regions of the country. In total, the Portuguese team conducted 53 semi-structured interviews from April to December 2019. A total of 38 were conducted in person in several regions of the country, and 15 were performed by video call. The author conducted 18 interviews with LGBTQI+ individuals with ages ranging from 32 to 50 years old.

The analysis also draws on qualitative information gathered through the focus group with healthcare providers to elicit their reactions to excerpts of interviews with self-identified asexual people conducted within The Asexual Revolution research. Excerpts were chosen for their depiction of contact with healthcare services and/or providers, of discovering the concept of asexuality and how it made sense for the interviewees, and of how interviewees navigated their intimate relationships.

The group format allowed for interaction amongst peers and thus created some ease that would not have been there in in-depth interviews. This process tends to eliminate the effects of observation and facilitates the perception of personal experiences as collective, minimising obstacles of excessive rationalisation or performance. The rationalisation, which is always present, becomes partially diluted in the perception of a casual discourse, emerging in the heat of the discussion between equals, sharing the individual’s responsibility with the group. Focus group discussion is a popular and recognised research technique, suitable for stimulating the views of service users in general [24] and health services in particular [25]. Focus groups are considered appropriate devices when the objective is to explain how people perceive an experience, facilitating the understanding of the human experience, which is not always possible through other methodologies. Discussion groups can also provide information on perceptions, feelings, and attitudes [24]. This methodology provides a data corpus that mirrors the social reality of a cultural group [26], by reflecting and retracting (at a micro level) a society and a history and allows for the observation of social interaction and investigation of nuances and complexities of participants’ attitudes and experiences [27].

All interviews and focus group results were explored according to a thematic analysis [28], which consisted of considering salient themes in a text at different levels, and thematic networks aiming to facilitate the structuring and depiction of these themes.

### 2.4. Ethical Considerations

CILIA LGBTQI+ Lives received ethical clearance by the Ethics Committee of the Centre for Social Studies—University of Coimbra (Ethics Commission Report dated 21 April 2019). The doctoral research ‘The Asexual Revolution: discussing human rights through the lens of asexuality in Portugal’ was approved by an internal panel of experts of the host institution, followed by the acceptance by the Scientific Committee of the Centre for Social Studies—University of Coimbra, taking into account good research practices in ethical terms in Portugal.

## 3. Results

### 3.1. Life Course Inequalities

The view of Portugal as a country of great legislative advances and significant improvements in the lives of LGBTQI+ people was apparent to almost all interviews of the CILIA LGBTQI+ Lives project. Interviewees made it clear that despite this progressive legal context, they felt insecure and little protected in various spheres of life, such as the workplace [29], services, healthcare, and especially on the street. For this fear to manifest, there is no need for verbal or physical abuse. Glares, lack of acknowledgement in the workplace, having others assume they are heterosexual, and invisibility in public representations (from popular media to institutional posters and brochures at the doctor’s office) are seen as indicative of homophobia or transphobia. The interviewees also felt a growing presence of conservative discourses in the public sphere, such as psychologists and psychiatrists advocating for conversion therapies or far-right slogans defended in social networks and Parliament. All of this leads to restraining affectionate gestures in public and the constant self-policing of body and verbal language [30].

The following quote exemplifies how heterosexuality is assumed:

*What I feel is that sometimes the health professional assumes our [heterosexual] identity. And there are certain resources that he does not make available because he starts off from this assumption. And I think that there are specific health issues related to sexual intercourse between gay men and I think that doctors are still not aware of that. And then there is that slightly confusing moment when we have to put it on the table and… (…) It’s more these things, these assumptions, these social assumptions of who we are and the impact that this has on the resources that we have access to or not. (Isaac, cis gay man 30–34 years old).*

However, at the same time, the opinion that the situation in Portugal has improved for LGBTQI+ people was consensual to almost all interviewees. They make it clear that rapid legal advances, from the approval of same-sex marriage onwards, are seen as an important safety net. LGBTQI+ people recognise that law has a pedagogical role, considered to be mostly symbolic, because they feel that mentalities have not kept up with these advances and that in socio-cultural terms there is still a lot to do.

For example, Mariana, a cis lesbian woman, describes the experience she had at the hospital while in labour:

*When I went to the hospital, I said ‘look, I’m a lesbian, this is my son’s other mother, my partner, her name is Maria’. And the healthcare professional asked ‘but, you know, here everyone is a husband (…) so how do I say it? Partner?’. And I said ‘yes, you can say partner which is a beautiful word, or you can simply say Maria’ (…) So, the lady went outside and said ‘[mispronounces name]’s partner can come in’. [laughs]. The lady got all red in the face and came inside and said (…) I was so confused that I didn’t even know how to say your name. (Mariana, cis lesbian woman, 45–49 years old).*

Although Mariana describes this episode as a positive experience, for the healthcare provider asked her about the best way to act in that situation, it nevertheless reveals that the healthcare professional had a great difficulty in acting beyond the established norms.

Participants in the CILIA LGBTQI+ Lives knew that they enjoyed a privileged setting in Portugal, but they also thought the country is still very homophobic and transphobic, so they mainly stay in the closet. They recognised that they are allowed to report situations of violence, prejudice and discrimination. However, reporting does not bring practical implications, nor do they feel confident in the training of police authorities and health personnel.

### 3.2. Focus Group with Healthcare Providers

Considering the most prominent aspects of the session conducted for The Asexual Revolution project, the focus group results were grouped in three major outcomes. Firstly, old tropes about a lack of sexual interest that were present in the professionals’ reaction to the testimonies are addressed. Next, the author considers narratives of willingness to learn about the subject that include the revaluation of clinical cases they had come across in their professional career. Finally, constructive and encouraging aspects concerning the healthcare providers’ discourse towards asexuality are highlighted, as well as its importance in contributing to the respect for human sexual diversity. These moments did not occur separately throughout the focus group and are not attached to the discourses of all the healthcare providers. The intention is to analyse biomedical discourse as a whole and not by their specific area of expertise; therefore, participants in the focus group are identified as HP (Healthcare Provider) followed by a number and not by their area of expertise.

#### 3.2.1. Old Tropes at the Doctor’s Office

The narratives produced in the focus group made evident harmful interpretations of asexuality done by professionals. This situation happened by explicitly verbalising it, by making jokes about the asexually identified people’s words (reacting with statements such as: “If she is a Carmelite nun, she’s doing fine!”), or by commenting on it in a subtler way through ambiguous statements of attempts to co-opt discourse on sexuality (minorities trying to ostracise majorities). The consensus amongst healthcare providers was that asexually identified people were building up barriers around themselves, were giving too many explanations and justifications about their intimate biography and behaviours, and were blocking other possibilities of experiencing life. Their interpreting actions and behaviours as pathological led necessarily to the search of an aetiology. Numerous attempts to find the causes to trace the origin of the behaviour of people who identify as asexual were made throughout the discussion. For example, certain types of personality were considered be propensive to certain ways in which people experienced their sexuality and narrated their intimate biography. Discredit by professionals and the dismissal of complaints as “that is all in your head” encountered in the focus group were corroborated in the testimonies of asexually identified participants.

*I once went to the gynaecologist and told her “it hurts a little bit when I do it’, ‘ah, this must be all in your head”. And she gave me pills and more pills and I was like “no, it hurts, it really hurts”, “maybe it’s your partner”. And it was not. Now, I realize that maybe it was me, because I felt so forced, it was almost as if I were raped. Daniela.*

Within the different explanations of “asexual behaviour”, the psychoanalyst approach went further in the explanation and often made a clear connection between the relationship of each of these people with their parents (present/absent or the search for these references in other people) and the building of barriers as responsible for the “development” of asexuality in more than one interviewee. For example, this comparison with a case encountered at the practice earlier was particularly telling:

*I recall a girl, who is the daughter of very well educated local people, that looked me up when she was 21 and that felt absolutely no pleasure. At 15, she had declared that she was a lesbian, but I think it was more of a bomb that she wanted to plant! (…) Then, she would go to clubs and mess around with one guy or another. Then, when psychotherapy started she was one of the hardest cases. (…)… but, meanwhile, I suggested family counselling. And the thing is, in the meantime she met some guy, started feeling pleasure, and attraction and love, I mean, the whole package. HP4*

*She normalized, so to say. HP2*

HP4′s description of this case reveals that identifying as a lesbian was interpreted as an act of rebellion against her family and that the absence of pleasure, considered to come with it, a source of distress. After searching for psychoanalytical help and engaging in family therapy, she “normalized” (to use the words conveyed by the other focus group participant, HP2) her intimate journey with “the full package”—engaging in a mono-hetero-relationship with a man she had felt attracted to, had fallen in love with and that (most likely) gave her pleasure.

Healthcare providers believed there was an apparent correlation between a good integration of sexuality in life and enduring or permanent relationships. Several short-lived relationships in a row or polyamorous constellations (e.g., expressions such as “several at the same time”, “do they have group sex?”) were equated as an inability to establish affectionate mature relationships or to manage the lack of attraction while presenting themselves in a normative way to society, and so it was stated that the partner “goes outside [that relationship], to his other partners to find what she cannot give him” and that “afterwards, to society, they introduce themselves as if everything is fine”.

When confronted with an account of asexuals exploring diverse sexual behaviours and practices with different sexes until finding recognition in the concept of asexuality, the healthcare providers classified it as a search for a normative relationship, namely, when they mentioned they wished to someday become parents. Asexuality was then considered to be a self-imposed (although unconscious) blockage to the development of a considered normal sexuality.

*I notice some illiteracy here. She does not go looking for help, does not know how to. Does not appreciate specialized help. Does not want to. She went online, searched for three or four things and made her own decisions. HP1*

This statement speaks to the idea that knowledge resides on what this focus group participant called “specialized help”, meaning medical knowledge in his perception. 

#### 3.2.2. Narratives of Willingness to Learn

As excerpts of the interviews with asexually identified people were being discussed during the focus group, it was possible to observe the genuine interest of most of the focus group participants in understanding more about asexuality and in the narratives of people who identified within the spectrum. Namely, by profusely taking notes of aspects that interviewees described and which they found important, and asking questions about facets—“What is [name LGBT youth organization]? Is it just online?”, “Did you also see/talk to *asexuated* men?”—and terminology—“This grey area. I did not know the concept. I don’t know if you [addressing the other focus group participants] did?”; “What does kinky mean?”—that they did not know about and wanted to comprehend better.

Numerous times, healthcare providers showed familiarity with some of the experiences narrated in the testimonies collected through interviews. When asked about direct interaction with asexuals at their office, all declined (knowing that they were) having contact with people who identified as asexual, but were beginning to find similarities with cases and stories—and “sexual behaviours”—discussed at appointments that now made sense to place within this identity/orientation.

*And her speech [referring to a client] was this one, she felt romanticism, she felt some attraction, she thought she wanted to have children, but she got there [sexual intercourse], full stop. I was listening to you read this case and it could be exactly what that patient told me in several appointments. I had even placed the hypothesis of vaginismus, but no, not with her, it was completely impossible, if he [partner] approached her for intercourse, she simply refused. It could be one of these [cases], I do not know. HP2*

Narratives showing a willingness to learn were also prevalent:

*I started to look back and eventually try to figure out, from thousands of situations that I had seen, if there were situations that fit within this type of case. And I think so, I think so. (…)*

*There were situations related to desire, especially to desire, which were situations of this kind. Because there was no personal suffering except when pressed. (...)And the pressure was social too. For example, with the internet, someone with whom that person could identify, right? And we have always been accustomed to people being constantly asking us, “Oh, doctor, is this normal?” HP5*

More controversial opinions within the group were also disputed. HP1′s construing of one case as illiteracy was quickly rebutted, and others in the meeting elucidated him that the interviewee had not “asked for clinical help” for reasons she had stated plainly in her interview:

*Because she is afraid of the medicalization of the situation. (…) Of being labelled as sick. HP3*

*I think it is very significant that she has the perception that her problem is not clinical, she has that perception. HP5*

*The gynaecologist did not have an appropriate behaviour. HP2*

*No, she did not allow that another specialist could come in (...) HP1*

*I mean, she [the doctor] buried her deeper “there, you go to the bottom. It’s his fault!” HP2*

Or HP1′s focusing on age as a synonym of immaturity and the direct connection to the fact that the sample of research participants was very young. Rapidly, other healthcare providers invalidated his affirmations, stating that the recruitment for this research was performed through the internet and that type of strategy is both inescapable and leads to this type of sample. Therefore, asexuality was not directly connected to young people or to sexual immaturity. The disproval of HP1′s take on activism as a “modern contamination to be socially considered more interesting” also showed the recognition of people’s subjective—and often hybrid—experience, and of the strategies they found to make it visible and claim for rights. 

#### 3.2.3. A Constructive and Encouraging View of Asexuality

Asexuality has become more visible in Portugal in recent years, following what has been happening in other regions in the West, through intense phases of online activity and media inclusion. That became evident throughout the discussion in the focus group, when some professionals made clear that they had heard the term asexual and other identities in the spectrum, such as demisexual [31]. In that respect, this exchange is very telling:

*(…) I have great difficulty in saying that this woman is asexual. (…) Let’s see, “I only feel sexual attraction after meeting the person more deeply”. “When I have an emotional connection”, it’s a bit like the feelings and the physical part are beginning to relate. For me, this description, which is a frequent description, many people identify with this description, right? (…) I think the demisexuals… and this is a case…*

*The what? HP2*

*Demisexuals. HP5*

*Oh, demisexuals! I heard that, it was Machado Vaz. HP2*

*This description is of a demisexual.* *HP5*

*(…)*

*I heard Professor Machado Vaz.* *HP2*

*But it fits the description, I would say, of the women of my time [laughter]. HP5*

*Exactly! Of our time, almost of mine as well, exactly. HP2*

*When what was allowed to them…HP5*

*Was modesty. HP2*

*What was allowed to them in terms of sexuality was that it appeared after emotional involvement. HP5*

In parallel, from the reports collected in the interviews, one can infer that the acceptance by healthcare professionals of the self-identified asexuals’ experiences and feelings is seen as comforting and a source of relief, detaching from themselves the burden of guilt and responsibility.

## 4. Discussion

LGBTQI+ people who have participated in the CILIA LGBTQI+ Lives project report a general perception of things improving for gender and sexually diverse people in Portugal. Interviewees believed that prejudice and intolerance have decreased in recent years, and that they feel more protected in terms of law and labour issues. Nevertheless, many confessed they avoid holding hands on the street, going to certain places, or taking certain routes, for fear of physical violence. This fact is consistent with the most recent data from The European Agency for Fundamental Rights [32], which reveal that although Portugal is considered to be a safe country, it is below average when it comes to being openly LGBTQI+.

Therefore, LGBTQI+ people in Portugal verbalised that there is still much to be done in their opinion. They pointed out the need for visibility in the public space, more representation and representativeness in public images and spaces (from mainstream media to institutional spaces) and, most of all, an urgency in changing mentalities. This necessary change in the way people face sexual and gender diversity will come from investing in the education of younger age groups and professionals of different sectors, such as the police and other law enforcers, and healthcare professionals.

For the healthcare providers that took part in the focus group, resorting to therapy meant that people were denying their identity or orientation. Gabriela Moita [13] refers to the interpretation of distress by homosexual users who resort to (or are sent to) therapy as a denial or request to change their sexual orientation. She considers this interpretation to be heterosexist. Heterosexism is a term proposed by Stephen Morin [17], meaning beliefs and attitudes that do not attach the same value to lifestyles of same sex people and people of different sexes. Generally, the term is used to refer to an ideological system that denies, denigrates, and stigmatises any non-heterosexual behaviour, identity, relationship, or community. Additionally, it is used to characterise heterosexual prejudices against homosexuals as well as behaviours based on these prejudices, which suggests a parallelism between anti-gay feelings and other forms of prejudice such as racism, anti-Semitism, or sexism. One of the pragmatic effects of heterosexism is the need for homosexual individuals to pass as heterosexual [33]. This same rationale can easily be used to address asexuality where the discourse (and pressure) is similar and the influence of therapists and health professionals in decisions about the users’ sexuality is significant. What led the participants in The Asexual Revolution project to seek counselling or medical advice was an attempt to get to know themselves better and try to understand the gap between what they felt and social expectations. This was interpreted by clinicians as suffering that necessitates the search for a diagnosis by therapists. Then, the healthcare provider will want to guide the process of discovering the client’s identity. This is an expression of the healthcare provider’s expectations and not of the client’s desire. This posture reveals a difficulty in dealing with and accepting the concept of asexuality, but also reveals heterosexism and sexucentrism [34], in the sense that it is accepted that the client’s well-being and their social adaptation depend on the adoption of certain established norms about sexuality.

Irrespective of the justification found for interviewees’ asexuality, behind this type of affirmation is the a priori view that there is a single adequate type of “learning sexuality” and that in the case of people that describe the absence of sexual attraction that learning was, at some point, inadequate. The healthcare providers tended to attribute the supposedly inadequate learning about sexuality to the “bad influence of the internet”, or a traumatic first relationship experience that people were (consciously or not) trying not to repeat. The answer to this was to search for specialised help that would accurately inform them about what they were feeling and frame it competently. Looking for medical advice and for information online, as some asexually identified people did, made one healthcare provider in the focus group conclude that the participant was illiterate in terms of health, which connects to the idea—also expressed by other professionals—that asexuality is related to or caused by inadequate types of learning. At the same time, it expresses a judgement that poses medical knowledge as superior to the embodied experience of the person: the doctor knows better, even if the client is the one that is having and living with the experience. This reveals more than lack of knowledge about asexuality as part of sexual diversity, but also an unconscious attempt of invalidation by framing it in an existing diagnosis (one that healthcare providers are trained to know)—either mental or physical. It implies, at the same time, a difference of value attributed to the experiences of asexual people (as being inferior). Within this framework, the attempt to find explanations for asexuality can send messages to the client that asexuality is pathological. The attempt to find causes for asexuality was maintained throughout the focus group, sustaining positions that understood asexuality as a result of learning factors—as in the case one interviewee’s house being small and the fact that she heard (and reacted to) her parents having sex—of psychobiological causes— “this is a clear case of dyspareunia”—or multifactorial causes, which end up creating a strong insistence on the pathologisation of asexuality.

In parallel (rather than alternatively), healthcare providers showed genuine interest in learning and requesting access to information to better welcome and respect the people who seek them and to give them the highest possible self-determination. After exploring rapport with terms belonging to the asexual lexicon and lived experience narrated in the interviews, it became clear that knowledge about asexuality which was starting to build up came from recent media coverage in national outlets with which the members had become familiar. Other health professionals that the author relied on throughout the research as privileged informants and bridge elements for access to certain spaces reported that they had used both the Facebook page *Assexuais em Portugal* as well as the doctoral project’s website [4] for information and links. They had shown both the platforms to users and told them “why don’t you check this out and see if it makes sense to you?”, leaving it to the client to assert the value of this information for their own life. Therefore, it is safe to say that the presence of asexually identified people in the media and their overall collective action resulted in messages being slowly apprehended by the general public and arriving, in a constructive way, at the doctor’s office.

By educating themselves on asexuality, healthcare professionals were able to understand that asexuality is a very diverse phenomenon. Healthcare professionals must therefore work with clients who seek their help or advice in terms of sexual disinterest to understand if asexuality makes sense for them. In other words, this means that healthcare professionals must learn about asexuality, the asexual community, and asexual activism, and be extremely aware of their clients reports of distress caused by social stigmatisation or pressure by their partner(s). Often, clients that seek them have not been introduced to the concept and to this reality, and trust healthcare providers to help them understand the ongoing changes they experience. By stating this, it does not mean that clinicians should assign identifications to those who seek their help. Rather, based on the data and analysis offered by empirical research, it seems crucial that the healthcare professional creates an environment in which the person can understand themselves and accept that asexuality is just one amongst a myriad of valid/available possibilities. The doctor’s office should be a safe space for sharing and discussing.

## 5. Conclusions

In conclusion, this article analysed how healthcare providers in Portugal perceive asexuality. From the inquiry, the author draws lessons that asexuality can teach us about the respect for gender and sexual diversity.

Both the literature on LGBTQI+ issues and healthcare and the data analysis revealed that healthcare providers express the need for cultural competence to better serve sexual and gender diverse people. Moreover, gender and sexual diversity appeared to be an unfamiliar topic to many healthcare providers, despite the growing visibility over the past years. They recognised that familiarising themselves with lived experience of asexual people helped them revaluate and better understand cases that they had come across in their journey. Additionally, in that process, the media had become a source for learning about asexual and LGTBQI+ experiences, which they can incorporate in their medical practice.

Therefore, cultural competence could enable them to interact more effectively with people from different communities, some of them in vulnerable positions. This means that the provided care must be informed and sensitive to be respectful and responsive to the health beliefs and practices of diverse groups, as well as to their cultural and linguistic needs, and knowledgeable of specific issues and demands. Awareness of sociocultural background and particular terminology is essential in providing the best care to LGBTQI+ clients. 

The results of the research that led to this article highlight the urgency of raising awareness and training of future generations of healthcare professionals in gender and sexual diversity. The inclusion of LGBTQI+ issues in several curricular units in healthcare Higher Education Institutions and Vocational Training, making it an integral aspect of discussing health is fundamental, and should go beyond workshops and seminars that are either optional or parallel to formal established curricula. Additionally, lifelong learning for healthcare professionals can help develop these competences. 

Considering the data collected in the focus group, as well as academic literature on the subject, activists’ claims and other empirical research, the author ultimately calls for the building of coalitions between groups and organizations advocating for sexual rights, sex education, and reproductive rights with sexual and gender minorities collectives, academics, the media and, of course, health students and professionals—to resist impositions of medicalisation and the pharmaceutical industry. In this act of claiming rights through resistance, asexual people should build strong bridges with other gender and sexual dissidents and other sexual minorities that are medically categorised alike. They can find common ground to collaborate on transforming the medical discourses imposed on sexuality.
